# An Available Strategy for Nasal Brain Transport of Nanocomposite Based on PAMAM Dendrimers via In Situ Gel

**DOI:** 10.3390/nano9020147

**Published:** 2019-01-24

**Authors:** Huichao Xie, Lingjun Li, Yue Sun, Yuzhen Wang, Shuang Gao, Yuan Tian, Xuemei Ma, Chengcheng Guo, Fumin Bo, Li Zhang

**Affiliations:** 1 College of Pharmacy, Shandong University of Traditional Chinese Medicine, Jinan 250355, China; m18366133344@163.com (H.X.); iamsy0404@163.com (Y.S.); wangyz1112@163.com (Y.W.); gaoshuang0315@163.com (S.G.); maxuemei1993@163.com (X.M.); gcc624474248@163.com (C.G.); a17862969519@163.com (F.B.); 17862969052@163.com (L.Z.); 2 College of Graduate, Shandong University of Traditional Chinese Medicine, Jinan 250355, China; 13854133712@163.com

**Keywords:** PAMAM dendrimer, nanocomposite, in situ gel, gellan gum, nasal brain transport

## Abstract

Polyamidoamine (PAMAM) dendrimers are efficient drug carriers. The presence of a physiological pathway for nasal brain transport provides a potential path for direct brain-targeted delivery of dendrimer nanocomposites. In this study, we synthesized PAMAM dendrimer composites with a nanoscale size; the particle size of PAE (Paeonol)/mPEG (the heterofunctional PEG polymer with a methoxy)-PAMAM G5.NHAc and mPEG-PAMAM G5.NH_2_-FITC were 72.41 ± 11.58 nm and 96.51 ± 7.77 nm, and the zeta potential of PAE/mPEG-PAMAM G5.NHAc and mPEG-PAMAM G5.NH_2_-FITC were + 0.57 ± 0.11 mv and + 9.60 ± 0.41 mv, respectively. The EE% and DL% of PAE in PAE/mPEG-PAMAM G5.NHAc were 53.77% and 13.92%, respectively. PAE/mPEG-PAMAM G5.NHAc/DGG ionic-sensitive in situ gel was prepared, the viscosity of solution and gel state were 112 ± 3.2 mPa and 1403 ± 38.5 mPa, respectively. The in vitro goat mucoadhesive strength of the gel was 4763.36 ± 85.39 dyne/cm^2^. In situ gel system was proven to be a non-Newtonian pseudo-plastic fluid with shear thinning, thixotropy and yield stress. The optimal model of PAE released from PAE/mPEG-PAMAM G5.NHAc and PAE/mPEG-PAMAM G5.NHAc/DGG were the Higuchi equation and the Korsmeyer-Peppas equation, respectively. The cytotoxicity of the nanocomposites showed a concentration-dependence, and the cell viabilities of PAE/mPEG-PAMAM G5.NHAc were both higher than 95% between 0.0001 μM and 10 μM. mPEG-PAMAM G5.NH_2_-FITC was efficiently taken up by cells and exhibited strong fluorescence in the cytoplasm and nucleus. Significant accumulation of nanocomposites was observed in the brain after administration of the in situ gel group, and maximum accumulation was reached at 12 h. A small amount of accumulation was observed in the nanocomposite solution group only at 2 h. Therefore, the direct nasal brain transport efficiency of PAMAM dendrimer nanocomposites can be significantly improved after combining with in situ gel. PAMAM dendrimer nanocomposite/DGG is a potential drug delivery system for nasal brain transport.

## 1. Introduction

The presence of the blood-brain barrier (BBB) and transmembrane efflux (TEE) makes it difficult to deliver therapeutic drug molecules to the brain region [1,2]. Brain-targeted drug delivery systems based on polyamidoamine (PAMAM) dendrimers with targeted modification have been successfully constructed more and more. PAMAM dendrimers are highly branched nano-sized polymer nanocomposites with ethylenediamine as the initial nucleus [3,4]. A large number of surface groups on the surface of dendrimers can be used for functional modification [5]. Surface electrostatic adsorption or bonding and physical entrapment of internal large hydrophobic cavities are effective forms of drug-loading by dendrimers [6]. Transferrin (Tf) and serine-arginine-leucine (SRL) were confirmed as brain targeting primers, which could effectively modify PAMAM dendrimers [7,8]. However, the brain-targeted delivery system of dendrimers modified by target proteins was unstable in vivo. Complex enzymatic, ionic, and pH environments posed challenges to the stability of the nanocomposites [9].

In recent years, the construction of nasal brain delivery systems has received increasing attention. Using PAMAM dendrimers as drug carriers to deliver drugs through the nasal brain transport for brain delivery has generated great interest. Compared with targeted drug carriers that enter the brain through the BBB by blood circulation, nasal brain transport delivers drugs directly to the brain via physiological pathways without targeted modification. In addition, nasal brain transport as a non-invasive method of administration will provide great convenience in the clinical application of drugs [10]. Nasal administration of PAMAM-loaded small interfering RNA (siRNA) resulted in florescence-tagged siRNA being found in the cytoplasm and processes of neurons and of glial cells in many brain regions, including the hypothalamus, amygdala, cerebral cortex, and striatum [11]. PAMAM-loaded haloperidol improved brain targeting after nasal administration, with 6.7 times lower doses of the dendrimer-haloperidol formulation administered via the intranasal route producing behavioral responses that were comparable to those induced by haloperidol formulations administered via an intraperito-neal injection [12]. The main limitation of nasal administration is the rapid clearance of drugs by nasal mucosa and cilia, which limits the adhesive time between the preparation and nasal mucosa [13]. In situ gel is a new type of environmentally responsive gel for nasal administration that changes from the solution state to gel state by responding to ions, temperature, or pH in the nasal environment [14]. The solution state before the gel phase transition maintains a stable low viscosity level, enables good fluidity of the preparation, and is convenient for drug administration and dosage control. The gel state formed after phase transformation has a higher viscosity level, which can adhere to the surface of the nasal mucosa and slow the clearance of the nasal cavity to increase the contact time and the absorption level [15]. In situ gels have been proven to be effective carriers for nasal brain transport of polymers. Youssef Naha [16] preparated novel nasal almotriptan loaded solid lipid nanoparticles in mucoadhesive in situ gel formulation for brain targeting. Mura [17] developed a liposomal mucoadhesive thermo-sensitive in situ gel for the extended nasal delivery of opiorphin. The ionotropic material deacetylated gellan gum (DGG) was selected as the matrix of the gel phase transition, which responded to Na^+^, K^+^, and Ca^2+^ in the nasal mucus. HMPC was used as a bioadhesive preparation. HPMC molecules were rich in hydroxyl groups, which formed hydrogen bonds with water molecules in the nasal mucosa, increased the adhesion strength between the gel and mucosa, and prolonged the cleared time of the gel. Mannitol was used as an isotonic agent, chlorine acetate was added as a preservative, and vitamin E was used as an antioxidant. 

Paeonol (PAE) was selected as a representative drug to study the drug loading and drug release ability of the drug delivery system. Previous studies demonstrated that PAE protected rat hippocampal neurons against oxygen-glucose deprivation-induced injury [18], attenuated cerebral ischemic injury in mice [19], and improved the behavior of Parkinson’s disease in animal models [20]. Oral administration, intravenous injection, and drug solution acting directly on model cells are the usual administration routes for PAE [21]. In summary, PAE is a potential central neuroprotective agent [22]. PAE is an active monomer isolated from the dried rhizome of *Paeonia Suffruticosa*. Its chemical structure is 2′-Hydroxy-4′-methoxyacetopheone, which is poorly water soluble [23]. However, PAE has a low volume of brain intake and a rapid metabolism in vivo with a half-life of only 5 min, leading to low bioavailability of the brain and difficulty in maintaining efficacy [24,25]. Increasing the amount of PAE into the brain is the key to improving bioavailability. 

The purpose of our study was to achieve nasal brain transport by combining functionally modified PAMAM dendrimer nanocomposites with in situ gel, and to study the physiochemical properties and biological effects. 

## 2. Materials and Methods 

### 2.1. Materials

Polyamidoamine dendrimers, generation 5, primary amine surface, and methanol solution (PAMAM G5.NH_2_) was purchased from Dendritech (Midland, Michigan, USA). The heterofunctional PEG polymer with a methoxy and succinimidyl ester functional groups (mPEG-NHS, Mw: 2000), fluorescein isothiocyanate (FITC), Phosphate buffer (pH 7.4), Borate buffer (pH 8.4), paeonol (PAE, >99%), and Hydroxypropyl methyl cellulose (HPMC, 30000 mPa·s) were purchased from Shanghai Yuanye Biological Technology Co., Ltd. (Shanghai, China). All other chemicals were purchased from Shanghai Yuanye Biological Technology Co., Ltd. (Shanghai, China), unless otherwise stated. Methylene Blue and Vitamin E were purchased from Solarbio (Beijing, China). Centrifugal filters (MW = 3000 Da and MW = 10 KDa) and dialysis bag (MW = 3500 Da) were purchased from Amicon (Darmstadt, Germany). (4,5-dimethylthiazol-2-yl)-2,5- diphenyltetrazo -lium bromide (MTT) and Deacetylated gellan gum (DGG) were purchased from Sigma-Aldrich (St. Louis, MO, USA). Wistar rats (200–220 g) were purchased from the Wuhan Sericebio Co., Ltd. (Wuhan, China) and were handled according to the Principles of Laboratory Animal Care. The protocols were approved by the Wuhan Sericebio Co., Ltd. (Wuhan, China). Animal Ethical Committee (NO.42000600030537).

### 2.2. Synthesis and Characterization of the PAMAM Dendrimer Nanocomposite

#### 2.2.1. Synthesis of the PAMAM Dendrimer Nanocomposites

##### Synthesis of mPEG-PAMAM G5.NH_2_

**Scheme 1:**
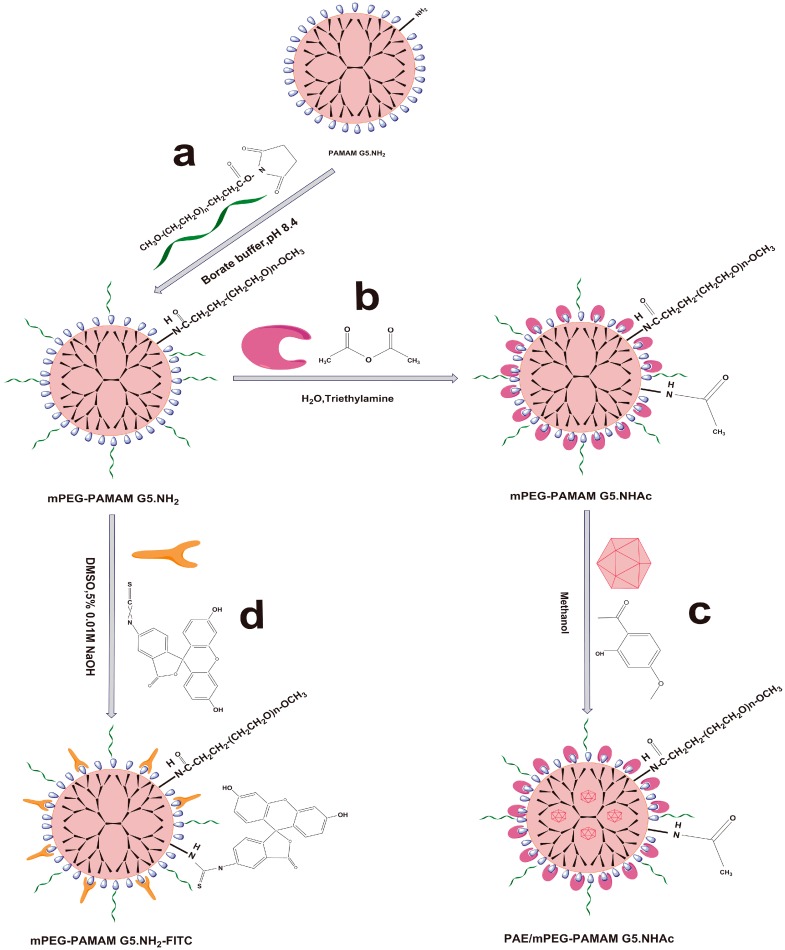
Synthesis schematics for the PAMAM dendrimer nanocomposites. (**a**) Synthesis of pegylated PAMAM dendrimer (mPEG-PAMAM G5.NH_2_); (**b**) synthesis of mPEG-PAMAM G5.NHAc by the addition reaction between anhydride and the amino group; (**c**) paeonol (PAE) loading in the cavities of mPEG-PAMAM G5.NHAc; (**d**) the FITC tracer was used to label PAMAM to observe the nasal brain transport of PAMAM dendrimer nanocomposite in vivo.

mPEG was coupled to the periphery of PAMAM G5.NH_2_ through the addition reaction of NHS and NH_2_ groups according to Scheme 1a. Briefly, PAMAM G5.NH_2_ (205.74 mg) was dissolved in 10 mL borate buffer (pH 8.4), then 10 mL borate buffer (pH 8.4) containing mPEG-NHS (148.71 mg) was added to the mixture under intense electromagnetic stirring. The reaction mixture was stirred under nitrogen for 48 h at room temperature and kept in the dark. Ultrafiltration centrifugation (MW = 10 KDa, 2000 rpm) was used to remove free mPEG-NHS and concentrated nanocomposite solution. The removal of free mPEG-NHS was confirmed by thin layer chromatography using a methanol-chloroform (2: 10 V/V) solvent system. We used lyophilization to prepare mPEG-PAMAM G5.NH_2_. FTIR and ^1^H-NMR were used to characterize the obtained compounds.

##### Synthesis of mPEG-PAMAM G5.NHAc

The amino groups on the surface of the PAMAM G5.NH_2_ were acetylated by the addition reaction between anhydride and amino groups, then the mPEG-PAMAM G5.NHAc macromolecule was synthesized as shown in Scheme 1b. Briefly, mPEG-PAMAMG5.NH_2_ (105.54 mg) was dissolved in distilled water (40 mL), triethylamine (160 μL) was added to the mixture under intense electromagnetic stirring, and stirring was continued for 30 min to keep the system weakly alkaline. Acetic anhydride (23.36 mg) was added dropwise. The reaction mixtures were stirred under nitrogen for 48 h at room temperature and kept in the dark. The reaction mixtures were purified and concentrated by Ultrafiltration centrifugation (MW = 10 KDa, 2000 rpm). The final products of mPEG-PAMAM G5.NHAc were frozen and lyophilized to remove water, and then characterized by FTIR and ^1^H-NMR.

##### Synthesis of mPEG-PAMAM G5.NH_2_-FITC

FITC was coupled to the periphery of PAMAM G5.NH_2_ through the addition reaction of isothiocyanato and NH_2_ groups according to Scheme 1c. Briefly, mPEG-PAMAM G5.NH_2_ (52.37 mg) and FITC (20.81 mg) were dissolved in 15 mL DMSO containing 5% 0.01 M NaOH under intense electromagnetic stirring. The reaction mixtures were stirred under nitrogen for 48 h at room temperature and kept in the dark. Ultrafiltration centrifugation (MW = 10 KDa, 2000 rpm) was used to remove free FITC and concentrated nanocomposite solution. mPEG-PAMAM G5.NH_2_-FITC was prepared by lyophilization. The reaction progress was confirmed by FTIR and ^1^H-NMR spectroscopy.

#### 2.2.2. Encapsulation of Paeonol into mPEG-PAMAM G5.NHAc

##### Preparation of PAE/mPEG-PAMAM G5.NHAc

Paeonol was physically loaded into the cavities of the mPEG-PAMAM G5.NHAc as described in Scheme 1d. Briefly, mPEG-PAMAMG5.NHAc (80.38 mg) was dissolved in 30 mL methanol, and 10 mL of methanol containing paeonol (19.39 mg) was added to the mixtures under vigorous electromagnetic stirring. The solution was protected from light and stirred for 48 h under nitrogen at room temperature. The reaction mixtures were further purified and concentrated by ultrafiltration (MW = 3500 Da, 2000 rpm). After lyophilization, PAE/mPEG-PAMAM G5.NHAc was obtained as a white solid. FTIR and ^1^H-NMR were used to characterize the product.

##### Drug Loading Efficiency and Encapsulation Efficiency

The drug loading efficiency and encapsulation efficiency of the PAE/mPEG-PAMAM G5.NHAc nanocomposite were determined by HPLC. The instrument used was an Agilent 1200 liquid phase system equipped with a UV-vis multi-wavelength detector. The column was Kromasil 100-5-C18 (4.6 × 250 mm, 5 μm), the mobile phase was methanol: water (60:40), the flow rate was 1 mL/min, the temperature was 30 °C, and the detection wavelength was 274 nm. The calibration curve for the paeonol concentration and absorption was Y = 100362.1012 X + 24.076, R^2^ = 0.9996. The ultrafiltrate containing free paeonol was collected and the concentration was determined by HPLC. The EE% (encapsulation efficiency %) and DL% (drug loading %) of PAE loaded in mPEG-PAMAM G5.NHAc were determined by the following equations:

(1) $$\begin{array}{l} EE\%=\frac{\mathrm{Amount}\;\mathrm{of}\;\mathrm{PAE}\;\mathrm{in}\;\mathrm{complexes}}{\mathrm{Total}\;\mathrm{amount}\;\mathrm{of}\;\mathrm{PAE}\;\mathrm{added}}*100\%\\ DL\%=\frac{\mathrm{Amount}\;\mathrm{of}\;\mathrm{PAE}\;\mathrm{in}\;\mathrm{complexes}}{\mathrm{Amount}\;\mathrm{of}\;\mathrm{complexes}}*100\% \end{array}$$

#### 2.2.3. Characterization of PAMAM Dendrimer Nanocomposites

##### FTIR

Solid samples of different PAMAM dendrimer nanocomposites were prepared by using dried KBr pellets under infrared light. FTIR spectra was obtained on a Fourier infrared spectrometer (Agilent 660-IR, California, USA) at wave number ranging from 4000 to 400 cm^−1^.

##### ^1^H-NMR

The structures of different PAMAM dendrimer nanocomposites were studied by proton Nuclear Magnetic Resonance (^1^H-NMR) (Bruker 600 MHz AVIII HD, DE). mPEG-PAMAM G5.NH_2_, mPEG-PAMAM G5.NHAc, mPEG-PAMAM G5.NH_2_-FITC, and PAE/mPEG-PAMAM G5.NHAc were dissolved in D_2_O at a concentration of 1 mg/mL, respectively, and the hydrogen spectra were obtained at a frequency of 600 MHz.

##### Particle Size and Zeta Potential

The particle size and zeta potential of PAMAM G5.NH_2_, mPEG-PAMAM G5.NH_2_, mPEG-PAMAM G5.NHAc, mPEG-PAMAM G5.NH_2_-FITC, and PA/mPEG-PAMAM G5.NHAc were determined using a dynamic light scattering (DLS) particle size analyzer (Zetasizer Nano ZS, Malvern Instruments Ltd, UK). The nanocomposites were dissolved in distilled water at a concentration of 1 mg/mL, respectively. Three independent measurements were taken for each sample to obtain the average particle size and zeta potential.

##### Transmission Electron Microscope (TEM) and Scanning Electron Microscope (SEM) 

The size and morphology of PAMAM G5.NH_2_ and PAE/mPEG-PAMAM G5.NHAc were observed by TEM (JEOL JEM-2100, Tokyo, Japan), with an acceleration voltage of 200 KV. The sample was dissolved in distilled water at a concentration of 0.1 mg/mL and a drop was placed on the carbon film, respectively. This was dried naturally for 12 h. PAE/mPEG-PAMAM G5.NHAc was dissolved in distilled water to 1 mg/mL, and then lyophilized. The surface of the sample was sprayed with gold to observe the morphology of it by SEM (Hitachi S-4800, Tokyo, Japan).

### 2.3. Preparation and Characterization of In Situ Gel

#### 2.3.1. Preparation of In Situ Gel

According to the method described by Jifu Hao [26], DGG was weighed accurately, dispersed in distilled water, heated to 90 °C with continuous stirring at 500 rpm and maintained for 30 min, and DGG was fully dissolved. After cooling to room temperature, the solution was fully swollen at 4 °C for 24 h. The HPMC was added to the DGG solution in batches under high speed agitation and swelled for 12 h. Other excipients were added under agitation, and stored at low temperature for later use.

#### 2.3.2. Response Surface Methodology

The effect of the independent variable on the response was studied by the central composite design (CCD) method. DGG concentration and HPMC concentration were two independent factors, and the dependent responses were the viscosity of the in situ gel solution (A) and the viscosity of the in situ gel (B). The experimental design was generated and evaluated by Design-Expert software (Stat-ease Inc., Minneapolis, MN, US). The in situ gel matrix (Table 1) was optimized using a two-factor and five-level (−Alpha, −1, 0, 1, +Alpha) full factorial design. According to the relationships between independent variables and responses described by CCD, the optimization area was set up, and the in-situ gel prescription containing PAE/mPEG-PAMAM G5.NHAc nanocomposites was formulated.

#### 2.3.3. Characterization of In Situ Gel

##### Viscosity

The viscosity of the in situ gel formulation was measured using a rotary viscometer (NDJ-8S, Shanghai, China). The measurement was carried out at 60 rpm using a No. 3 rotor, and the temperature was maintained at 34 °C. The viscosity was read directly from the viscometer display. All measurements were made in triplicate.

##### Critical Ionic Concentration (CIC)

Critical ionic concentration was an important parameter of ionic in situ gel, indicating the minimum ionic concentration that promoted phase transition. According to the method described by Sneha R. Salunke (IN, 2016) [27], simulated nasal fluid (SNF) was prepared: 8.77 g NaCl, 2.98 g KCl, and 0.59 g CaCl_2_ were dissolved in 1 L distilled water. Different volumes of artificial nasal fluid were mixed with 1 mL of in situ gel solution in an ampoule, respectively. After 30 s, the gels adhered to the bottom of bottles without flowing, indicating that the gels formed. The normal physiological temperature of the human nasal cavity is 32 °C–34 °C. All measurements were taken at 34 °C and made in triplicate.

##### Gel Strength

Gel strength was defined as a measure of the ability of a polymer dispersion to develop and remain in a gel form. The gel strength was determined according to the method described by Mahajan HS [28].The in situ gel of 50 g was moved precisely in a 100 mL measuring cylinder, and a 35 g weight was then placed on the top of the gel. The time required to move the piston 5 cm down through the gel was recorded. All measurements were taken at 34 °C and made in triplicate.

##### Water Holding Capacity

The water holding capacity of the gel was determined as stated by Hosny KM [29]. In situ gel (1 mL) was accurately transferred, and put in a centrifuged EP tube that had been accurately weighed. 250 μL of artificial nasal fluid was then added and mixed, respectively. The gel quality was accurately weighed and recorded as W_0_. This was centrifuged at 8000 rpm for 10 min, and the separate water layer was blotted by filter papers. The gel was weighed accurately and recorded as W. Water holding capacity = W/W_0_ × 100%. All measurements were taken at 34 °C and tested in triplicate.

##### Volume Expansion Coefficient

The volume expansion coefficient of the gel was determined as mentioned by Vidhi Shah [30]. In situ gel (1 mL) was removed accurately into a graduated test tube, 250 μL of artificial nasal fluid was added and mixed with it, and the volume was recorded as 1.25 mL. 2 mL artificial nasal fluid was added and then in situ gel was formed after phase transition; the final volume was recorded as V_T_. Therefore, the gel volume after phase transition was V_G_, V_G_ = V_T_ − 2.0. Each sample was tested three times at 34 °C. The expansion coefficient, S%, of the gel was described as:

(2) $$S\%=\frac{V_{G}-V_{M}}{V_{M}}*100\%$$

##### pH

The pH of the in situ gel solution was measured by using a digital pH meter (INESA PHS-3C, Shanghai, China). The digital pH meter was calibrated with pH 4.0 potassium hydrogen phthalate buffer and pH 6.86 mixed phosphate buffer solution, and the glass electrode was sufficiently dipped into the gel solution for measurement. Each measurement was tested three times at 34 °C.

##### TEM and SEM

The practice size of PAE/mPEG-PAMAM G5.NHAc loaded in situ gel was studied by TEM. A drop of in situ gel solution was deposited on the carbon film after dilution and dried naturally for 12 h. The morphology of in situ gel was studied by scanning electron microscopy. The in situ gel was lyophilized and then sprayed with gold for observation.

##### Rheology Analysis

The rheological properties of the gel before and after gelation were studied using a rheometer (Anton paar MCR302, Graz, Austria). All measurements were performed at 34 °C and examined three times.

The rheological curves before and after gelation were determined by setting the shear rate, γ (S^−1^), in the range of 0.01 to 1000 S^−1^. The sample was carefully placed into the sample pool with a spatula or key and sealed with silicone oil. After setting a gap of 0.1 mm, the shear stress, τ (Pa), and viscosity, η (Pa·S), was recorded, drawing τ = f (γ) and η = f (γ). 

The stress-strain curves before and after cementing were measured by setting the shear stress, τ (Pa), scanning range from 0.01 to 100 Pa, and the frequency (rotational speed) was set as 10 rad/s. The sample was carefully placed in the sample pool with a spatula or key and sealed with silicone oil. After setting a gap of 0.1 mm, the strain (%) and modulus (Pa), plotting strain (%) = f (τ) and modulus (Pa) = f (τ) were recorded.

Frequency scanning curves before and after gelation were determined by setting the frequency (Hz) scanning ranging from 0.01 to 10.00 Hz or speed (rad/s) ranging from 0 to 1000 rad/s. The sample was carefully placed in the sample pool with a spatula or key and sealed with silicone oil. The gap was set to 0.1 mm and strain to 1%. The modulus (Pa), drawing modulus (Pa) = f (Hz), calculating loss factor, tan δ, and drawing, tan δ = f (Hz), were recorded.

##### Nasal Mucociliary Transport Time

To compare the residence time of in situ gel and solution in the nasal cavity, we used the method described by Ahmed M. Fatouh to study the nasal mucociliary transport time [31]. Briefly, the physiological saline solution containing methylene blue dye at a concentration of 5 mg/mL and in situ gel with the same methylene blue content were prepared. Rats (n = 5) were anesthetized by intramuscular injection of sodium thiopental (7 mg/mL). 10 μL of each sample was infiltrated into the right nostril of the rat using a micropipette, respectively. The throats of the rats were wiped with wet cotton swabs, and the appearance time of the blue dye was recorded. Each sample was examined in triplicate.

##### In Vitro Mucoadsorption Strength

**Scheme 2:**
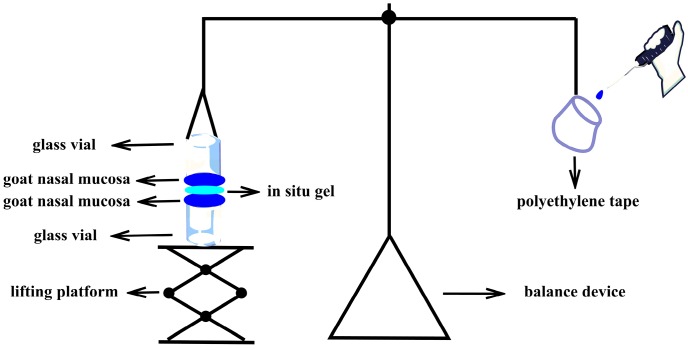
An improved physical balance method measuring in vitro mucoadsorption strength.

In vitro mucoadhesive strength was the force required to separate formulations from the nasal mucosa. The in vitro bioadhesion was measured by using an improved physical balance method described by Hitendra S. Mahajan (Scheme 2) [32]. Briefly, fresh goat nasal mucosa obtained from the local slaughterhouse was transported in PBS (phosphate baffered saline, pH 7.4) and used within 5 h. The nasal cavity was cut longitudinally along the nasal septum, and the nasal mucosa with a thickness of 0.6 mm was separated from the nasal olfactory area. The two mucosas were fixed to the openings of the two same glass vials by cyanoacrylate adhesive combined with thin wire; the mucosa side was outward, and the exposed area of the nasal cavity film was A (cm^2^). An empty polyethylene bag was connected at one end of the balance scale, and the bottom of one of the glass bottles was connected at the other end, with the bottle mouth downward. The mouth of another glass bottle was upward, as for the lifting platform, to balance gravity. The 50 mg gel was uniformly distributed on the mucous membrane of the glass bottle below, and the height of the lifting platform was adjusted to make the mucous membrane of the two glass bottles come into contact with the gel, and the balance was in the equilibrium position. A certain force was applied to make both sides of the mucous membranes be in contact with the gel for 2 min. Water was added dropwise to the empty polyethylene tape. The weight of the water (m) was recorded when the mucosa was separated from the gel. The experiment was performed in triplicate. The in vitro mucoadsorption strength was obtained by the following formula:

(3) $$\mathrm{Detachment}\;\mathrm{Stress}=\frac{m*g}{A}$$

where m is the weight added to the balance in grams; g is the acceleration due to gravity (taken as 980 cm·s^−2^); and A is the surface area of the mucosal tissue in cm^2^.

### 2.4. In Vitro Release

#### 2.4.1. Release of PAE from PAE/mPEG-PAMAM G5.NHAc Nanocomposite

**Scheme 3:**
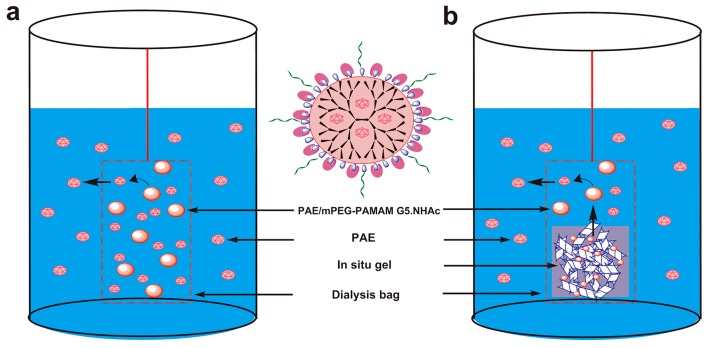
In vitro release devices of PAE from PAE/mPEG-PAMAM G5.NHAc nanocomposite (**a**) and PAE/mPEG-PAMAM G5.NHAc/DGG in situ gel (**b**). (**a**) PAE was released from PAE/mPEG-PAMAM G5.NHAc nanocomposite and spread evenly in the release medium (PBS, pH7.4); (**b**) PAE/mPEG-PAMAM G5.NHAc nanocomposite was released from in situ gel, then PAE/mPEG-PAMAM G5.NHAc nanocomposite released PAE.

The release of PAE from the PAE/mPEG-PAMAM G5.NHAc nanocomposite was investigated by the dialysis bag method (Scheme 3a). PAE/mPEG-PAMAM G5.NHAc was dissolved in PBS (pH 7.4), corresponding to PAE 1 mg/mL. 2 mL was accurately transferred and placed in a dialysis bag (MW = 3500 Da). The dialysis bag was placed in a release vial containing 25 mL of PBS (pH 7.4). The release experiment was carried out under a shaking condition of 100 rpm in a 37 ± 0.5 °C constant temperature oscillator. Samples of 1 mL were collected at 0.25 h, 0.5 h, 0.75 h, 1 h, 2 h, 4 h, 6 h, 8 h, and 12 h time intervals, and the same volume and temperature of the dissolving medium was replenished after each sampling. Samples were passed through 0.44 μm microporous membranes and determined by HPLC. Then, the cumulative release rate of PAE was calculated. Each measurement was tested at 34 °C. Experiments were repeated three times and the results were expressed as the mean values ± standard deviation (SD).

#### 2.4.2. Release of PAE from PAE/mPEG-PAMAM G5.NHAc/DGG In Situ Gel

The in vitro release study of PAE/mPEG-PAMAM G5.NHAc/DGG in situ gel was carried out by the dialysis bag diffusion technique (Scheme 3b). 1 mL PAE/mPEG-PAMAM G5.NHAc/DGG gel solution was mixed with 0.25 mL SNF to form in situ gel. In situ gel was placed in a dialysis bag (MW = 3500 Da) containing 5 mL of PBS (pH 7.4). The filled dialysis bag was immersed in a bottle containing 25 mL PBS (pH 7.4). The release was established at 100 rpm. Aliquots were withdrawn at 0.25 h, 0.5 h, 0.75 h, 1 h, 2 h, 4 h, 6 h, 8 h, and 12 h time intervals, and the same volume and temperature of the dissolving medium was replenished after each sampling. The PAE concentration was measured spectrophotometrically at 274 nm using HPLC. Each measurement was tested at 34 °C. Experiments were repeated three times and the results were expressed as the mean values ± standard deviation (SD).

#### 2.4.3. Release Kinetics

The release mechanisms of PAE from mPEG-PAMAM G5.NHAc nanocomposite and PAE/mPEG-PAMAM G5.NHAc/DGG in situ gel were studied by using zero-order, first-order, Higuchi and Korsemeyer-Peppa kinetic equations. The data was analyzed by Originpro 8.0 software (OriginLab, Massachusetts, USA).

### 2.5. Cell Evaluation

#### 2.5.1. In Vitro Cytotoxicity of PAMAM Dendrimer Nanocomposites

The cytotoxicity of PAMAM dendrimer nanocomposites were evaluated by the MTT method. The HepG2 cells in the logarithmic growth phase were inoculated into a 96-well plate at a density of 5 × 10^3^ cells/well, and cultured in a cell culture incubator (5% CO_2_, 37 ° C) for 24 h. 100 μL series concentration (0.0001 μM, 0.001 μM, 0.01 μM, 0.1 μM, 1 μM, 10 μM, and 100 μM) of PAMAM G5.NH_2_, mPEG-PAMAM G5.NH_2_, mPEG-PAMAM G5.NHAc, and PAE/mPEG-PAMAM G5.NHAc was added, respectively. Culture was continued for 12 h. The drug-containing culture solution was aspirated and washed twice with PBS. A serum-free medium containing 100 μL of MTT (0.5 mg/mL) was added to each well, and incubation was continued for 2 h at 37 °C. The culture was stopped, the culture medium was carefully sucked from the holes, 200 μL DMSO was added to each hole, and then the purple crystals were sufficiently dissolved by shaking in the dark. The absorbance value of each well at 492 nm was measured with a microplate reader, and the cell viability was calculated. Experiments were repeated three times.

#### 2.5.2. In Vitro Cellular Uptake 

Nanocomposites that could be taken up by cells was a prerequisite for absorption and transport. The HepG2 cells in the logarithmic growth phase were inoculated into a 96-well plate at a density of 1 × 10^4^ cells/well, and cultured in a cell culture incubator (5% CO_2_, 37 °C) for 24 h. 100 μL of 1 μM PAMAMG5.NH_2_- mPEG-FITC solution was added and incubated for 24 h at 37 °C. The medium was discarded, and washed 3 times with cold PBS (pH 7.4). Fluorescence and white sheets were recorded under a fluorescence microscope. All samples were tested in triplicate.

### 2.6. Fluorescence Evaluation of Nasal Brain Transport

Forty-five rats were divided into two groups randomly. One group was given 50 μL of mPEG-PAMAM G5.NH_2_-FITC/DGG solution to the unilateral nasal cavity, and one group was unilaterally administered 50 μL of mPEG-PAMAM G5.NH_2_-FITC solution to the nasal cavity. After administration of 0 h, 2 h, 6 h, 12 h, and 24 h time intervals, the rats were anesthetized by intraperitoneal injection of urethane, respectively. The heart was perfused with physiological saline to remove residual blood from the brain, and fixed with 4% paraformaldehyde. The head was cut off, the fur was removed, and the whole brain was separated. The tissue was fixed in 10% formalin (pH 6.2) at 4 °C. The in vitro brains were observed by an in vivo imaging system, and the fluorescence images of each group were recorded.

### 2.7. Statistics Analysis 

Data are presented as mean standard deviation. One-way analysis of variance (ANOVA) was used to determine significance among groups. All in vitro experiments were done in triplicates and the results are presented as mean ± standard error of mean. Differences were considered statistically significant at *p* ≤ 0.05. The *p* values of ≤ 0.05, ≤ 0.001, and ≤ 0.0001 are marked in figures with a single asterisk (*), double asterisk (**), and triple asterisk (***), respectively.

## 3. Results and Discussion

### 3.1. Synthesis and Characterization of PAMAM Dendrimer Nanocomposites

#### 3.1.1. Characterization of Synthesis by Spectrum

mPEG-PAMAM G5.NHAc was prepared by the addition reaction of NHS and acid anhydrides with the NH_2_ groups on the surface of the dendrimers, respectively. PAE was physically encapsulated in the cavities of mPEG-PAMAM G5.NHAc nanocomposite and then drug-loaded PAE/mPEG-PAMAM G5.NHAc nanocomposite was obtained. Similarly, mPEG-PAMAM G5.NH_2_-FITC nanocomposite was synthesized by coupling the surface NH_2_ groups and the isothiocyanates of FITC, which made the PAMAM dendrimer nanocomposite fluorescent.

**Figure 1:**
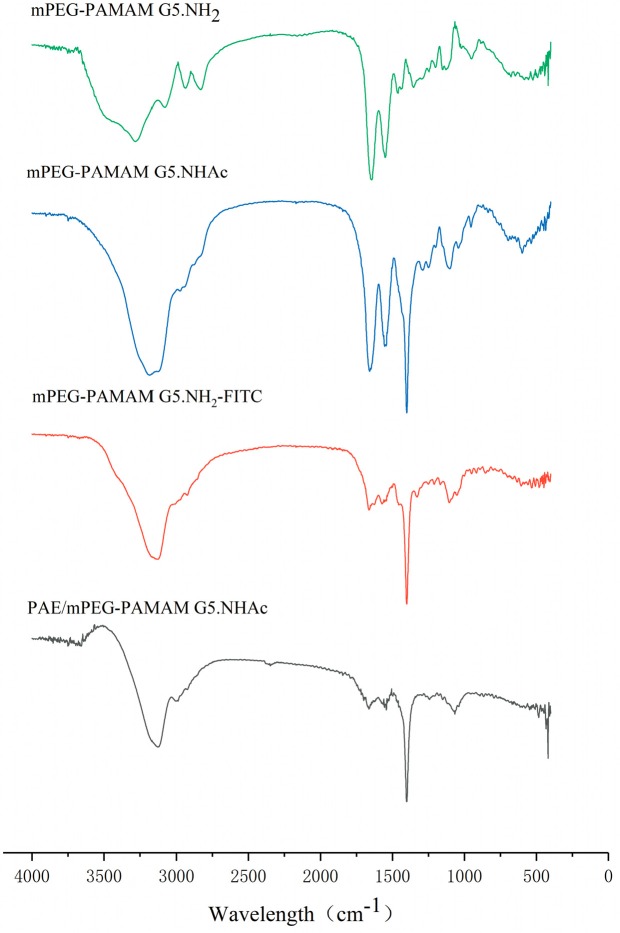
FTIR spectra of mPEG-PAMAM G5.NH_2_, mPEG-PAMAM G5.NHAc, PAE/mPEG-PAMAM G5.NHAc, and mPEG-PAMAM G5.NH_2_-FITC.

The successful syntheses of mPEG-PAMAM G5.NH_2_, mPEG-PAMAM G5.NHAc, and mPEG-PAMAM G5.NH_2_-FITC were confirmed by both FTIR and ^1^H-NMR of the nanocomposites. The infrared spectra of nanocomposites are shown in Figure 1. In the infrared spectrum of mPEG-PAMAM G5.NHAc, 3123.41 cm^−1^ and 1661.55 cm^−1^ were the stretching vibration of N-H and the stretching vibration of C = O of the amide in PAMAM G5.NH_2_. 1114.94 cm^−1^ and the fingerprint area near it were characteristic peak groups of ether groups in mPEG-NHS, and the two characteristic absorption peaks produced by carbonyl coupling of anhydride compounds in 1850 cm^−1^–1750 cm^−1^ disappeared. The information above indicated the successful synthesis of mPEG-PAMAM G5.NHAc. In the infrared spectrum of mPEG-PAMAM G5.NH_2_-FITC, the stretching vibration of the benzene ring skeleton in the range of 1650 cm^−1^–1430 cm^−1^ and the stretching vibration of hydrogens on benzene ring at 3000 cm^−1^–3100 cm^−1^ proved the existence of the benzene ring. Moreover, the characteristic absorption peaks of FITC’s S = C = N cumulated double bonds did not appear at 2039.66 cm^−1^. The above demonstrated that FITC was successfully bonded to the surface of PAMAM dendrimers.

**Figure 2:**
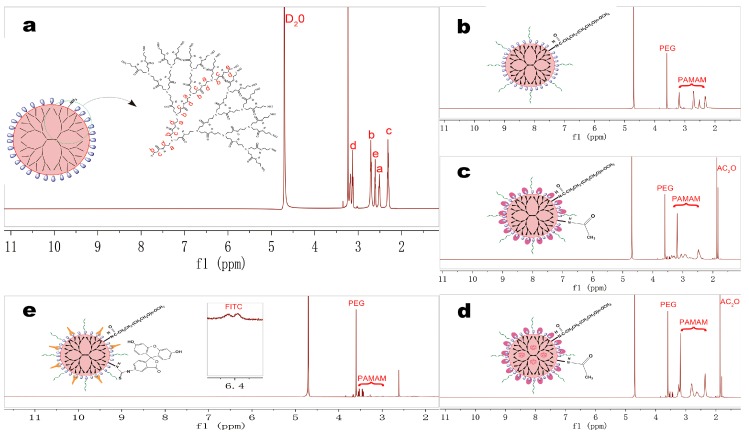
^1^H-NMR spectra of PAMAM dendrimer nanocomposites were obtained at 1 mg/mL concentration in D_2_O on a 600 MHz NMR spectrometer. (**a**) PAMAM G5.NH_2_; (**b**) mPEG-PAMAM G5.NH_2_; (**c**) mPEG-PAMAM G5.NHAc; (**d**) PAE/mPEG-PAMAM G5.NHAc; (**e**) mPEG-PAMAM G5.NH_2_-FITC.

The hydrogen spectra of the nanocomposites are shown in Figure 2. The spectrum of mPEG-PAMAM G5.NHAC is shown as Figure 2b, δ = 2.48 ppm belonged to -NH(C=O)CH_2_- in PAMAM G5.NH_2_, and δ = 3.60 ppm belonged to -CH_2_CH_2_O- repeating units in mPEG-NHS, δ = 1.82–1.90 ppm was the characteristic proton peaks of acetic anhydride (Figure 2c). The characteristic peaks corresponding to the aromatic ring of FITC were found in the spectrum of mPEG-PAMAM G5.NH_2_-FITC between δ = 6.30–7.90 ppm (Figure 2e).

#### 3.1.2. Particle size, Zeta Potential, and Morphology

The particle size and zeta potential of nanocomposites were determined by DLS and are shown in Table 2. The particle size of PAMAM G5.NH_2_ was consistent with the description [33]. After PEGylation, the particle size increased. PEG was a long chain molecule, but entangled on the surface of PAMAM after bonding. The degree of shrinkage in water was greater, so the particle size increased to a lesser extent. After acetylation of the remaining surface amino groups, the size of the nanocomposite increased significantly (*** *p* < 0.0001), which might be due to the high degree of acetylation and changes in the contraction of PEG chains. The gradual increase in the particle size during the bonding process also indirectly proved that the synthesis of nanocomposite was successful. The particle size of PAE/mPEG-PAMAM G5.NHAc showed no significant difference compared with mPEG-PAMAM G5.NHAc (*p* > 0.05), which indirectly proved that paeonol was encapsulated into internal cavities rather than bonded to the surface. 

The main cause of cytotoxicity was the positive charge of 128 primary amino groups on the surface of PAMAM G5.NH_2_ [34]. The zeta potential of nanocomposites was determined, with a range of + 0.57 mV to + 8.23 mV. After PEGylation, the zeta potential of mPEG-PAMAM G5.NH_2_ was reduced, possibly due to the shielding effect of PEG. The zeta potential of mPEG-PAMAM G5.NHAc prepared by acetylated modification was decreased significantly (* *p* < 0.05). It was interesting that the zeta potential of PAE/mPEG-PAMAM G5.NHAc was decreased further. The reduction of the zeta potential indirectly demonstrated that the bonding of the PAMAM dendrimer nanocomposite was successful.

**Figure 3:**
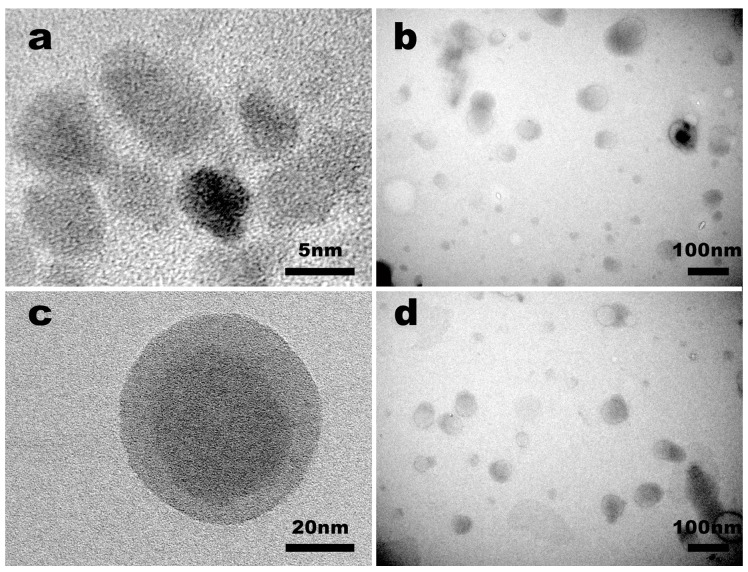
Transmission electron microscopy (TEM) images of nanocomposites. (**a**) PAMAM G5.NH_2_; (**b**) PAE/mPEG-PAMAM G5.NHAc; (**c**) high resolution image of a single PAE/mPEG-PAMAM G5.NHAc; (**d**) the image of PAE/mPEG-PAMAM G5.NHAc based DGG (deacetylated gellan gum) in situ gel.

**Figure 4:**
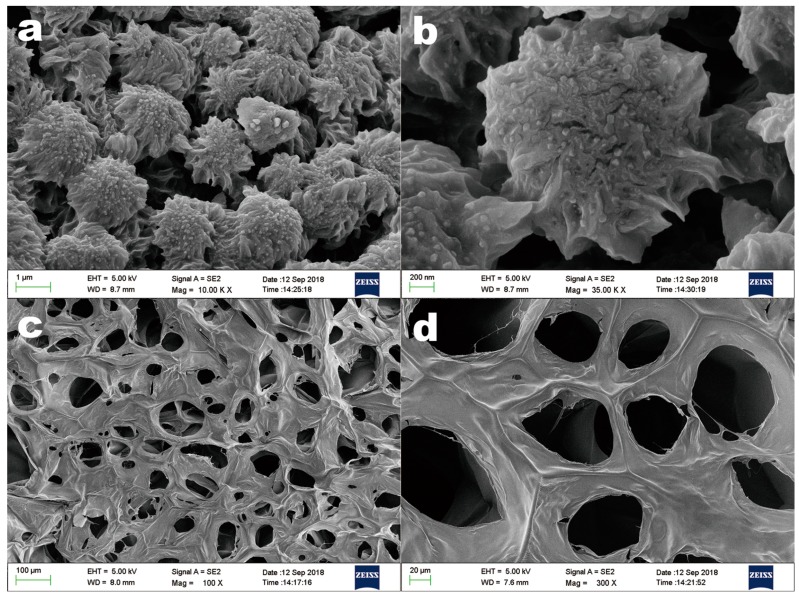
Scanning electron microscopy (SEM) images of the lyophilized PAE/mPEG-PAMAM G5.NHAc (**a**,**b**) and in situ gel (**c**,**d**).

The morphology and size of PAMAM G5.NH_2_ and PAE/mPEG-PAMAM G5.NHAc were observed by TEM (Figure 3). The results indicated that the size range of PAMAM G5.NH_2_ and PAE/mPEG-PAMAM G5.NHAc were 4 nm to 6 nm in diameter and 65 nm to 85 nm in diameter, respectively, in agreement with the results of DLS (dynamic light scattering) measurements. PAMAM G5.NH_2_ and PAE/mPEG-PAMAM G5.NHAc were both spherical. The morphology of PAE/mPEG-PAMAM G5.NHAc lyophilizate was observed by SEM (Figure 4). The size of the spheres was larger than that of PAE/mPEG-PAMAM G5.NHAc observed by DLS and TEM, probably due to the agglomeration of nanocomposites during lyophilization.

#### 3.1.3. EE% and DL%

The EE% and DL% of PAE in PAE/mPEG-PAMAM G5.NHAc were determined by HPLC, and were 53.77% and 13.92%, respectively. The three-dimensional cavities formed by the branched chains of PAMAM effectively loaded water-insoluble small molecule drugs. The long chains of PEG entangled on the surface of the dendrimers achieved the purpose of solidifying drugs [35].

### 3.2. Preparation and Characterization of In Situ Gel 

#### 3.2.1. Response Surface Methodology

**Figure 5:**
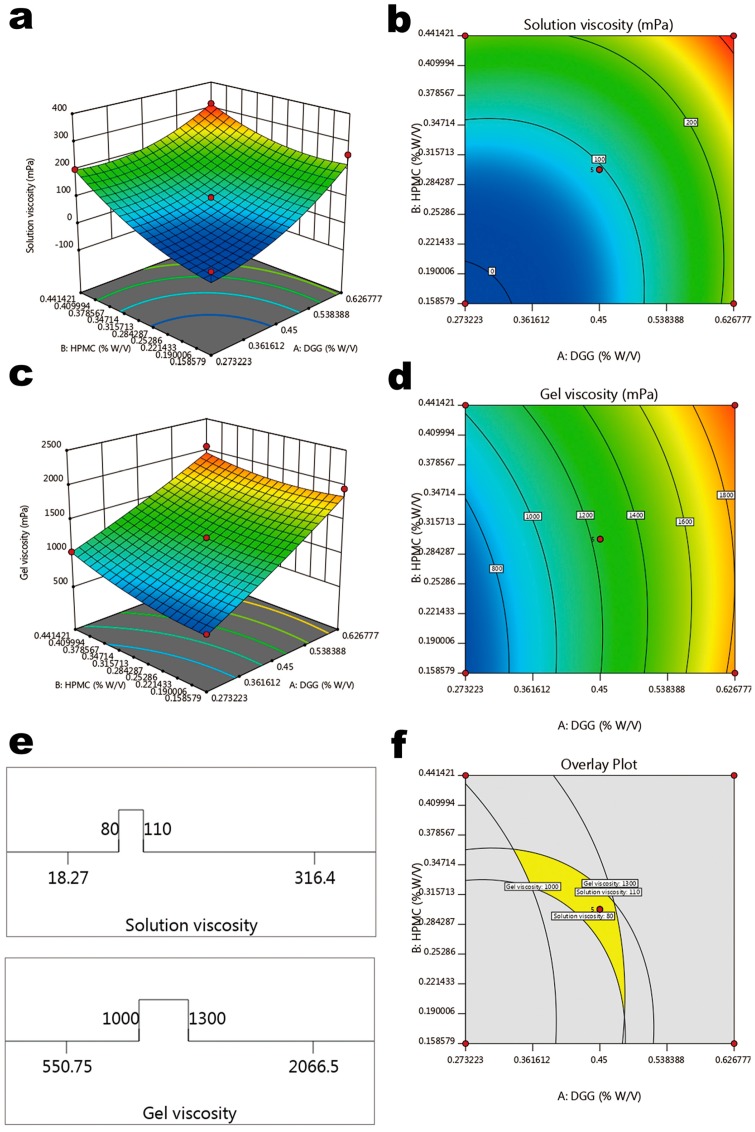
Three-dimensional surfaces and contours showing the influence of DGG and HPMC. (**a**) 3D surface plot of DGG and HPMC on solution viscosity; (**b**) contour of DGG and HPMC on solution viscosity; (**c**) 3D surface plot of DGG and HPMC on gel viscosity; (**d**) contour of DGG and HPMC on gel viscosity; (**e**) target value ranges of solution viscosity and gel viscosity; (**f**) graphical optimization highlighting an area of operability.

Table 3 shows the test run design matrix generated by the software (design expert) and the response values of the dependency factors. Figure 5 shows the 3D response surface and contour plots of the DGG concentration (A) and HPMC concentration (B) to the viscosity of the gel solution and gel. As shown in Table 4, based on the experimental results, the empirical relationship between the independent variables and the responses were shown using a second-order polynomial equation with coded values. The R^2^ values of the two fitting equations were all greater than 0.96, which proved that there were good correlations between the independent variables and the responses. The viscosity of the solution varied from 18.27 to 316.4 mPa, and the viscosity of the gel ranged from 550.75 to 2066.5 mPa. The viscosity of the solution increased gradually with the increase of the DGG concentration and HPMC concentration.

Similarly, the viscosity of the gel also increased gradually. Viscosity was an important parameter of in situ gel preparation. In situ gel solution should have a low viscosity, as a lower viscosity gives the solution a better flow behavior and a convenient way of administration. The gel formed after gelation should have a suitable viscosity range. It should have sufficient viscosity to increase the adhesion time between the gel and nasal mucosa, and to prolong the absorption time of the drug. However, excessive viscosity can cause cleaning difficulties. Figure 5f provides an optimized region of the DGG concentration and HPMC concentration. Taking values in this area enabled the formulation to have a lower viscosity in the solution state to meet the needs of drug delivery, and a suitable viscosity in the gel state provided a guarantee for the morphology of the gel and the adsorption strength of the mucosa.

According to the optimized results, 0.45% DGG and 0.3% HPMC were selected as the final concentration. Table 5 shows the optimized formulation of the PAE/mPEG-PAMAM G5.NHAc/DGG in situ gel.

#### 3.2.2. Characterization of In Situ Gel

Our experimental results showed that stable gels could be formed within 30 s for per 1 mL of PAE/mPEG-PAMAM G5.NHAc/DGG requiring 125 μL SNF. The requirement of a low critical ionic concentration shortened the phase transition time of the gel and a loss of the gel was prevented. Viscosity measurements indicated that the viscosity of the PAE/mPEG-PAMAM G5.NHAc/DGG solution was 112 ± 3.2 mPa, and the viscosity of the gel state was 1403 ± 38.5 mPa. The viscosity increased significantly after gel phase transformation (*** *p* < 0.0001), which established a formulation basis for increasing the adhesion time of the mucosa. Under normal physiological conditions, the nasal cilia oscillated at a rate of 1000 times/min [36]. The particles adhering to the surface of the nasal cavity migrated to the nasopharynx, then to the esophagus, and finally into the gastrointestinal tract [37]. The gel strength was generally in the range of 25 to 50 s, which resisted the removal of nasal cilia to protect the integrity of the gel, and ensured the sustained release of the drug at specific absorption site, especially suitable for nasal administration [32]. The gel strength of PAE/mPEG-PAMAM G5.NHAc/DGG was 28 ± 3 s, which was suitable for nasal administration. An excellent water-holding capacity (95.21% ± 1.58%) was beneficial to maintain the stability of the gel when stored or subjected to external forces. Due to the limited volume of the human nasal cavity, an excessive gel volume expansion coefficient led to nasal discomfort, so it was necessary to study the S% of the gel [30]. A volume expansion coefficient of 5.28% ± 0.41% was allowed. The pH of the nasal mucus was generally between 5.5–7.0 [38]. The pH of the in situ gel should be similar to the pH of the nasal mucus, so as to reduce irritation to the nasal mucosa. The pH of the PAE/mPEG-PAMAM G5.NHAc/DGG solution was 6.17 ± 0.17. A higher level of mucosal adhesion indicated that the gel could remain in contact with the mucosa for a longer period of time, increasing drug absorption. However, excessive adhesion caused difficulties in the removal of gel from the nasal cavity.

The in vitro mucoadhesive strength (4763.36 ± 85.39 dyne/cm^2^) after the phase change of PAE/mPEG-PAMAM G5.NHAc/DGG was accepted. The time to reach the nasopalatine and pharynx after intranasal administration of methylene blue solution was 1.8 ± 0.2 min and 8.6 ± 0.6 min, respectively. The time to reach the nasopalatine and pharynx after nasal administration of PAE/mPEG-PAMAM G5.NHAc/DGG was 34.7 ± 1.2 min and > 60 min, respectively. The nasal clearance time of PAE/mPEG-PAMAM G5.NHAc/DGG was significantly prolonged.

Figure 3d illustrated the state of which PAE/mPEG-PAMAM G5.NHAc was mixed into the in situ gel solution. The nanocomposite remained in a spherical form and dispersed in the in situ gel solution, and the particle size (60 nm–85 nm) was not affected. The gelation state of the in situ gel is shown in Figure 4c. Abundant cations shielded the electrostatic repulsion in molecular chains of the gellan gum, under the action of hydrogen bonds, polymerization and crosslinking of the double-helix dimer in DGG were promoted, and a three-dimensional network structure was formed [39].

#### 3.2.3. Rheology Analysis

**Figure 6:**
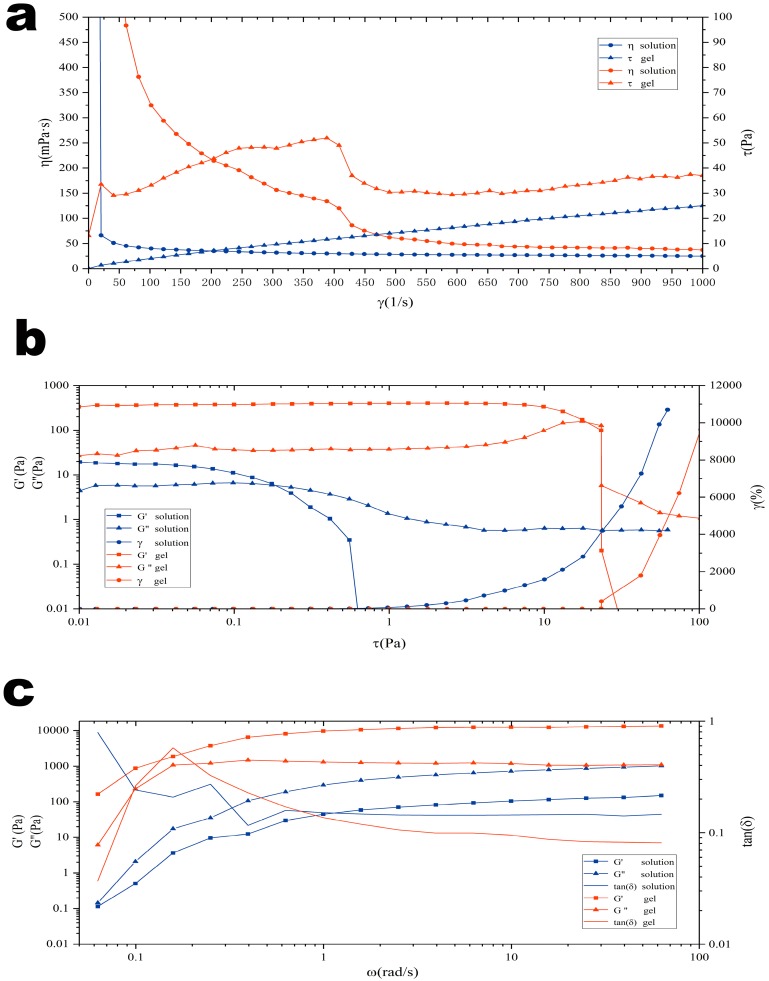
Rheological evaluation of the DGG in situ gel with PAE/mPEG-PAMAM G5.NHAc at 34 °C. (**a**) Rheology characterisitics of the DGG solution and the DGG in situ gel; (**b**) stress strain of the DGG solution and the DGG in situ gel; (**c**) frequency sweep of DGG solution and the DGG in situ gel.

The evaluation of rheological properties was an important part of nasal administration preparations [40]. Appropriate rheological properties ensured that the gel reached the olfactory area of the nasal mucosa and maintained a longer period of time, increasing the drug absorption time [41]. Figure 6 depicts the shear rate curves, frequency sweep curves, and stress-strain curves before and after gelation of the in situ gel. The results showed that the solution was a Newtonian fluid with a stable viscosity and good fluidity. Additionally, it was proven that the gel was a non-Newtonian pseudoplastic fluid with shear thinning, thixotropy, and yield stress.

In the shear rate curve (Figure 6a), the viscosity of gellan gum solution did not change with the increase of shear frequency, but the viscosity of the gel was gradually decreased, so it could be judged that the in situ gel after gelation was a non-Newtonian mechanics pseudoplastic fluid. This characteristic of the gel helped to maintain a low viscosity state under the oscillating shear of the nasal cilia, increasing fluidity, thus making it easier for gel to reach the deeper olfactory zone and facilitating the release of nanocomposite in the olfactory zone.

The frequency sweep curve (Figure 6b) describes the relationship between gel viscoelastic properties and time scale. The storage modulus (G″) was used to evaluate the elasticity of samples, and the loss modulus (G′) was used to evaluate the viscosity of samples. The relative relationship between the two determined the properties exhibited by the samples. In the solution, both increased linearly with the change of angular frequency scanning. The loss modulus (G′) > storage modulus (G″) indicated that the in situ gel solution was a viscous fluid, and good fluidity was convenient for administration. When the artificial nasal fluid was mixed to form gel, the storage modulus (G″) > loss modulus (G′) of the system, which showed that the elasticity was the dominant property of the gel, indicating the formation of a gel network spatial structure after gelation.

Figure 6c depicts the stress-strain curve of the solution and gel. Both the loss modulus (G′) and the storage modulus (G″) of the solution and gel remained stable at low strain conditions, indicating that the gel had a stable three-dimensional network space structure. When the stress was further increased, the storage modulus (G″) of the gel decreased rapidly, indicating that the elasticity of the gel was lowered, the spatial structure was destroyed, and the gel had shear thinning, thixotropy, and yield stress.

### 3.3. In Vitro Release

**Figure 7:**
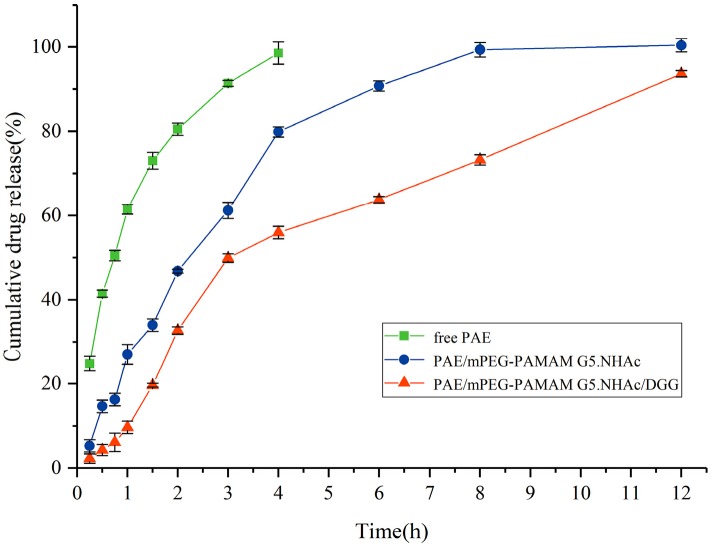
In vitro release profiles of PAE from PAE/mPEG-PAMAM G5.NHAc nanocomposites and PAE/mPEG-PAMAM G5.NHAc/DGG in situ gel in PBS (pH 7.4) at 37 °C. (Error shown as one standard deviation of the mean, n = 3).

The log P of PAE was 2.054 [42], and the saturation solubility was 557.69 mg/mL in pH 7.4 PBS (34 °C). The in vitro release tests assured sink conditions. The results of PAE released from PAE/mPEG-PAMAM G5.NHAc and PAE/mPEG-PAMAM G5.NHAc/DGG are shown in Figure 7. The release rate of free PAE at 4 h was 98.56% and the release was complete. The release rate of PAE from PAE/mPEG-PAMAM G5.NHAc was 79.90% at 4 h, and it was completely released at 12 h. The release of free PAE was significantly faster than that of PAE/mPEG-PAMAM G5.NHAc, confirming the sustained release effect of the nanocomposite on PAE. Drugs were wrapped by the PAMAM dendrimer nanocomposite, and the release process from cavities to the external medium effectively prolonged the elimination half-life of drugs in vivo.

The release of PAE from PAE/mPEG-PAMAM G5.NHAc/DGG was less in the first hour, and its release rate was low. The release rate increased after 1 h. The above results indicated that the release of PAE from PAE/mPEG-PAMAM G5.NHAc/DGG involved two consecutive processes: The release of PAE/mPEG-PAMAM G5.NHAc from the in situ gel and the release of PAE from PAE/mPEG-PAMAM G5.NHAc. In situ gel formed after phase transition had a stable three-dimensional network structure, which provided a pathway for PAE/mPEG-PAMAM G5.NHAc to release from in situ gel.

We used kinetic models to illustrate the release mechanism of PAE. Table 6 shows optimal models of the in vitro release. It was indicated that the release mechanism of PAE from the dendrimer nanocomposite was diffusion. The optimal model of PAE released from PAE/mPEG-PAMAM G5.NHAc/DGG indicated that the release mechanism was non-Fickan diffusion, and the release behavior was synergistic effects of drug diffusion and matrix erosion.

### 3.4. Cell Evaluation

#### 3.4.1. In Vitro Cytotoxicity of PAMAM Dendrimer Nanocomposites

**Figure 8:**
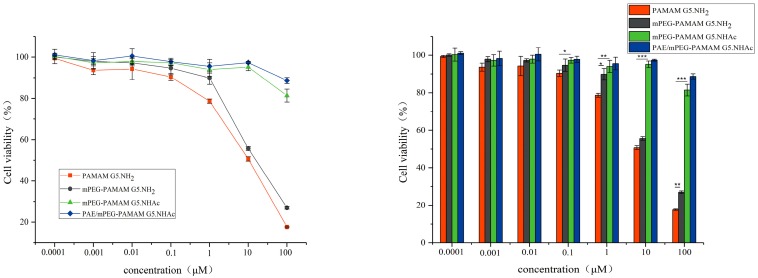
Cell viabilitiy of HepG2 cells following treatment with PAMAM G5.NH_2_ (0.0001–100 μM), mPEG-PAMAM G5.NH_2_ (0.0001–100 μM), mPEG-PAMAM G5.NHAc (0.0001–100 μM), PAE/mPEG-PAMAM G5.NHAc (0.0001–100 μM) for 12 h. (Error shown as one standard deviation of the mean, n = 3, * *p* < 0.05, ** *p* < 0.001, *** *p* < 0.0001).

Cytotoxicity was one of the important evaluation criteria for the biocompatibility of materials [43]. The main source of cytotoxicity in dendrimers was the positive charge carried by the 128 primary amino groups on the surface [44]. Negatively charged cell membranes interacted with primary amino groups, causing adsorption to each other, leading to the rupture and dissolution of cell membranes. The cell viability of different PAMAM dendrimer nanocomposites after 12 h of incubation are shown in Figure 8. The cytotoxicity was in turn PAMAM G5.NH_2_ > mPEG-PAMAM G5.NH_2_ > mPEG-PAMAM G5.NHAc > PAE/mPEG-PAMAM G5.NHAc. The cytotoxicity of nanocomposites showed a concentration-dependent effect, and the cell viability decreased with the increase of the concentration of nanocomposites. When the concentration was less than 0.1 μM, the cell viabilities of different nanocomposites were more than 90%, showing no cytotoxicity. The cytotoxicity of mPEG-PAMAM G5.NH_2_ was lower than that of PAMAM G5.NH_2_, because the entanglement of dendrimers by PEG chains reduced the exposure of amino groups on the surface. The cytotoxicity of mPEG-PAMAM G5.NH_2_ (* *p* < 0.05) and mPEG-PAMAM G5.NHAc (** *p* < 0.001) were significantly different from that of PAMAM G5.NH_2_ at the concentration of 1 μM. When the concentration was between 0.0001 μM and 10 μM, the cell viabilities of mPEG-PAMAM G5.NHAc and PAE/mPEG- PAMAM G5.NHAc were both higher than 95%, showing good biocompatibility. The elimination of cytotoxicity of mPEG-PAMAM G5.NHAc and PAE/mPEG- PAMAM G5.NHAc was due to the fact that excess amino groups on the dendritic surface were sufficiently acetylated and the surface positive potential decreased significantly. A single intranasal administration by low-dose treatment (3 μg) of PAMAM G4 dendrimers appeared to be not toxic, and a high-dose treatment (15 μg) may potentially lead to neuronal effects by modulating the gene expression of the brain-derived neurotrophic factor signaling pathway [45]. Our study indicated that acetylation modification significantly reduced the toxicity of PAMAM, but the relationship between the estimated concentration of nanocomposites needed to administer the therapeutic drug dose and toxicity should be further studied. 

#### 3.4.2. In Vitro Cellular Uptake 

**Figure 9:**
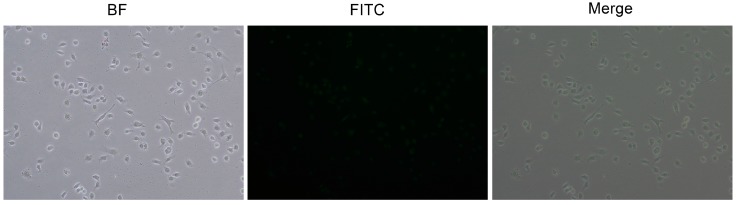
The uptake and localization of mPEG-PAMAM G5.NH_2_-FITC in HepG2 cells. The cellular uptake of dendrimer nanocomposites exhibited strong fluorescence in the cytoplasm and nucleus.

Figure 9 shows the uptake and localization of mPEG-PAMAM G5.NH_2_ -FITC in HepG2 cells observed by confocal microscopy. mPEG-PAMAM G5.NH_2_-FITC was efficiently taken up by cells and exhibited strong fluorescence in the cytoplasm and nucleus.

### 3.5. Fluorescence Evaluation of Nasal Brain Transport

**Figure 10:**
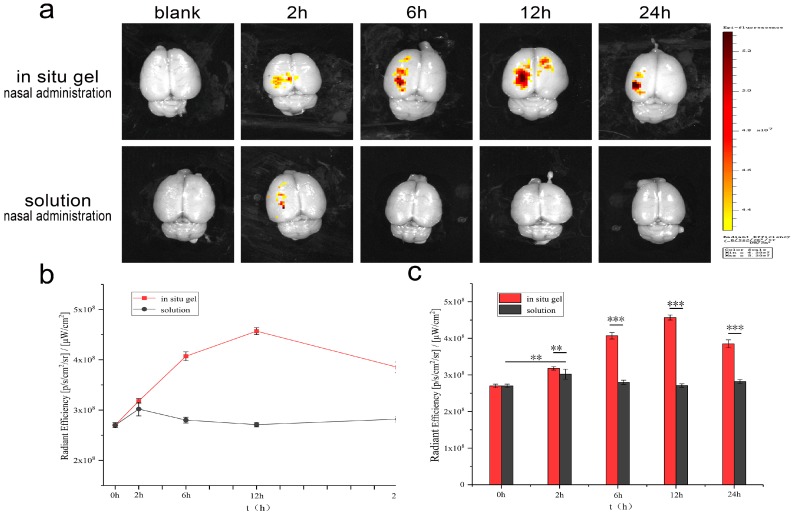
Fluorescence imaging of an isolated brain by the in vivo imaging system. The brain of 0 h is a blank control. The blank brain of rats has a matrix fluorescence response at 492 nm, and the fluorescence images at each time are the fluorescence response after eliminating the blank interference. (**a**) Fluorescence response in isolated brains; (**b**)The trends of fluorescence response of the solution group and the in situ gel group; (**c**)The difference of fluorescence response between the solution group and the in situ gel group. (Error shown as one standard deviation of the mean, n = 5, * *p* < 0.05, ** *p* < 0.001, *** *P* < 0.0001).

Figure 10 shows the fluorescence evaluation of nasal brain transport. In the nanocomposite solution group, only a small amount of accumulation was observed in the brain at 2 h after nasal administration. Significant accumulation of nanocomposites was observed in the brain after administration of the in situ gel group, and the maximum accumulation was reached at 12 h. The in situ gel group still had much accumulation at 24 h, indicating that the nanocomposites achieved nasal brain transport and were effectively retained in the brain, which was beneficial to achieve long-circulation effects of drugs. The radiant efficiency of the composite solution group and the in situ gel group was significantly different at 2 h (** *p* < 0.001), and the maximum radiant efficiency of the two groups was also significantly different (*** *p* < 0.0001), indicating that the in situ gel effectively increased nasal absorption and nasal brain transport of the nanocomposites. After nasal administration, the nanocomposite solution could not be effectively retained in the nasal cavity, and the dendrimer nanocomposites were cleared to the pharynx without being effectively released from the solution. Nasal administration of mPEG-PAMAM G5.NH_2_-FITC/DGG solution could form gel after phase transformation by responding to Na^+^, K^+^, and Ca^2+^ in the nasal mucus and then adhering to the nasal mucosa. The high viscosity level and gel strength of the in situ gel effectively slowed down the clearance of nasal mucus and cilia, providing sufficient time for the release of dendrimer nanocomposites from the gel and the absorption of the nasal mucosa. The single-molecule nanomicelle properties enabled PAMAM dendrimer nanocomposite to be absorbed and transported by the nasal cavity. After combining with in situ gel, the transport efficiency of nanocomposites can be significantly improved. These results indicate that PAMAM dendrimer nanocomposite/DGG is a potential drug delivery system for nasal brain transport.

The nasal epithelium had high permeability, and pathways of drug absorption through the nasal mucosa mainly included the lipid channels of cells and the aqueous channels between cells [17]. The pathways for nasal brain transport were mainly the systemic pathway and the nervous system. Drugs were absorbed by respiratory mucosa into the systemic circulation and subsequently reached the brain by crossing the BBB [46]. The neural pathway was composed of the nasal olfactory mucosa, the trigeminal nerve, and the olfactory nerve [47]. After being absorbed by the olfactory mucosa, drugs were directly delivered into the brain along the neural pathway. The single molecule nanomicelle property was beneficial to the transmission of the PAMAM dendrimer nanocomposite in the neural pathway [48,49,50]. The distribution of mPEG-PAMAM G5.NH_2_-FITC in the brain region, the transport capacity quantified over time in the brain and different brain regions, and the pharmacokinetics of PAE/mPEG-PAMAM G5.NHAc in the brain and different brain regions will be studied further.

## 4. Conclusions

PAMAM dendrimers were functionally modified and encapsulated paeonol in cavities. Ionic-sensitive in situ gel of dendrimer nanocomposites could be prepared successfully by using gellan gum. In situ gel was proven to be a non-Newtonian fluid with shear thinning, thixotropy, and yield stress. After nasal administration, the gel solution changed to a gel state by phase transition, and the bioadhesion was improved. In situ gel prolonged the release time and enhanced mucosal absorption. PAMAM nanocomposite solution has a low nasal absorption level, and in situ gel can significantly improve the nasal transport efficiency of nanocomposites. The PAMAM dendrimer nanocomposite/DGG delivery system becomes a potential nasal brain transport system of drugs.

## Figures and Tables

**Table 1:** Factor and levels in the optimization of in situ gel.

Factor	Minimum −1.414	Coded Low −1	Mean 0	Coded High +1	Maximum +1.414
A: DGG (% *w*/*v*)	0.2	0.2732	0.45	0.6278	0.7
B: HPMC (% *w*/*v*)	0.1	0.1586	0.3	0.4414	0.5

**Table 2:** Particle size and zeta potential of PAMAM dendrimer nanocomposites.

Compound	Particle Size (nm)	Zeta Potential (mv)
PAMAM G5.NH_2_	5.41 ± 0.24	+8.23 ± 0.55
mPEG-PAMAM G5.NH_2_	11.55 ± 0.14	+4.81 ± 0.17
mPEG-PAMAM G5.NHAc	70.91 ± 11.59	+2.60 ± 0.02
PAE/mPEG-PAMAM G5.NHAc	72.41 ± 11.58	+0.57 ± 0.11
mPEG-PAMAM G5.NH_2_-FITC	96.51 ± 7.77	+9.60 ± 0.41

**Table 3:** Central composite design experiments and experimental results.

Runs	Factor1 A:DGG (% *w*/*v*)	Factor2 B:HPMC (% *w*/*v*)	Factor1 A:DGG (% *w*/*v*)	Factor2 B:HPMC (% *w*/*v*)	Reponse 1 Solution viscosity mPa	Reponse 2 Gel viscosity mPa
1	−1	−1	0.2732	0.1586	22.83 ± 0.17	639.35 ± 3.89
2	−1	−1	0.6278	0.1586	254.21 ± 0.52	1955.02 ± 11.56
3	1	1	0.2732	0.4414	200.92 ± 0.58	1029.63 ± 10.35
4	1	1	0.6278	0.4414	316.43 ± 0.88	2066.50 ± 22.59
5	−1.414	0	0.2000	0.3000	45.67 ± 0.04	550.75 ± 8.67
6	1.414	0	0.7000	0.3000	294.50 ± 0.94	1945.54 ± 19.36
7	0	−1.414	0.4500	0.1000	18.27 ± 0.08	1167.21 ± 9.83
8	0	1.414	0.4500	0.5000	286.24 ± 0.77	1554.32 ± 9.93
9	0	0	0.4500	0.3000	100.50 ± 0.67	1243.51 ± 9.30
10	0	0	0.4500	0.3000	95.96 ± 0.82	1214.59 ± 7.82
11	0	0	0.4500	0.3000	97.92 ± 0.31	1240.45 ± 11.05
12	0	0	0.4500	0.3000	91.34 ± 0.53	1233.56 ± 9.39
13	0	0	0.4500	0.3000	96.01 ± 0.66	1250.27 ± 12.83

**Table 4:** Regression equations of the fitted models.

Reponse Y	Model	Regression Equation	F-Value	P-Value	R^2^
Y1:Solution Viscosity (34 °C)	Quadratic	Y1 = +96.33 + 87.35 × A + 77.40 × B − 28.97 × A × B + 46.23 × A^2^ + 37.31 × B^2^	35.75	<0.0001	0.9623
Y2:Gel Viscosity (34 °C)	Quadratic	Y2 = +1235.90 + 540.63 × A + 131.13 × B − 69.69 × A × B + 35.69 × A^2^ + 91.87 × B^2^	76.38	<0.0001	0.9820

**Table 5:** Compositions of in situ gel.

Ingredients	Formulation Compositions (% *w*/*v*)
PAE/mPEG-PAMAM G5.NHAc	1
DGG	0.45
HPMC	0.3
Mannitol	1
Chlorine acetate	0.01
Vitamin E	0.01
Distilled water	100

**Table 6:** Release mechanism of PAE.

Compound	Model	Regression Equation	R^2^
PAE/mPEG-PAMAM G5.NHAc	Higuchi	Q_t_ = 0.43459t^1/2^ − 0.16452	0.98
PAE/mPEG-PAMAM G5.NHAc/DGG	Korsmeyer-Peppas	Q_t_ = 0.18095t^0.68301^	0.95
